# Genetic Knock-Ins of Endogenous Fluorescent Tags in RAW 264.7 Murine Macrophages Using CRISPR/Cas9 Genome Editing

**DOI:** 10.21769/BioProtoc.4960

**Published:** 2024-03-20

**Authors:** Beverly Naigles, Jan Soroczynski, Nan Hao

**Affiliations:** 1 Deparment of Molecular Biology, University of California San Diego, La Jolla, CA, USA; 2 Laboratory of Genome Architecture and Dynamics, The Rockefeller University, New York, NY, USA; 3 Department of Bioengineering, University of California San Diego, La Jolla, CA, USA

**Keywords:** RAW 264.7, Macrophage, CRISPR knock-in, Endogenous tagging, Genome editing

## Abstract

CRISPR/Cas9 genome editing is a widely used tool for creating genetic knock-ins, which allow for endogenous tagging of genes. This is in contrast with random insertion using viral vectors, where expression of the inserted transgene changes the total copy number of a gene in a cell and does not reflect the endogenous chromatin environment or any trans-acting regulation experienced at a locus. There are very few protocols for endogenous fluorescent tagging in macrophages. Here, we describe a protocol to design and test CRISPR guide RNAs and donor plasmids, to transfect them into RAW 264.7 mouse macrophage-like cells using the Neon transfection system and to grow up clonal populations of cells containing the endogenous knock-in at various loci. We have used this protocol to create endogenous fluorescent knock-ins in at least six loci, including both endogenously tagging genes and inserting transgenes in the Rosa26 and Tigre safe harbor loci. This protocol uses circular plasmid DNA as the donor template and delivers the sgRNA and Cas9 as an all-in-one expression plasmid. We designed this protocol for fluorescent protein knock-ins; it is best used when positive clones can be identified by fluorescence. However, it may be possible to adapt the protocol for non-fluorescent knock-ins. This protocol allows for the fairly straightforward creation of clonal populations of macrophages with tags at the endogenous loci of genes. We also describe how to set up imaging experiments in 24-well plates to track fluorescence in the edited cells over time.

Key features

• CRISPR knock-in of fluorescent proteins in RAW 264.7 mouse macrophages at diverse genomic loci.

• This protocol is optimized for the use of the Neon transfection system.

• Includes instructions for growing up edited clonal populations from single cells with one single-cell sorting step and efficient growth in conditioned media after cell sorting.

• Designed for knocking in fluorescent proteins and screening transfected cells by FACS, but modification for non-fluorescent knock-ins may be possible.

**Graphical overview**


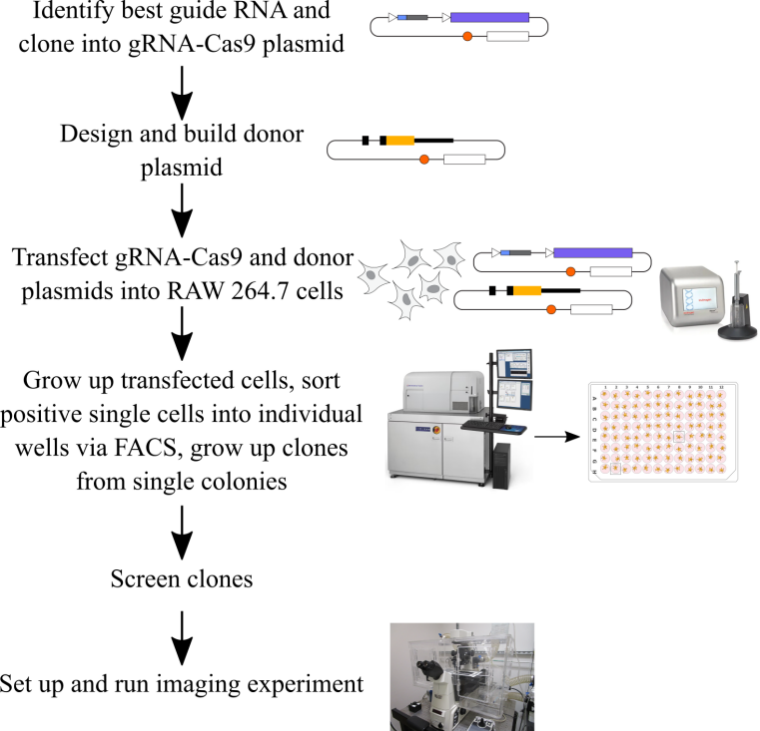


## Background

The use of CRISPR/Cas9 genome editing to insert DNA into the genome at a specific locus using the cell’s endogenous homology-directed repair (HDR) pathway is a valuable approach for modifying the genome of a cell [1,2]. One specific use of this technology is to endogenously tag genes with fluorescent proteins, enabling the study of gene expression and protein localization in live single cells. In contrast to the use of viral vectors to randomly insert transgenes into the genome, endogenous tagging ensures that the tagged protein is expressed using all endogenous regulation, including any trans-acting regulatory factors, which is useful for studying the mechanisms of gene expression. Endogenous tagging also ensures that the copy number of the protein remains the same in tagged and untagged cells, in contrast to the overexpression that occurs when using transgenes. There are existing knock-in protocols for many common easy-to-transfect cells lines, such as human RPE1, HCT116 [3], HeLa, and U2-OS cells [4], human iPSCs [5], and mouse embryonic stem cells [6]. In macrophages, there are existing protocols for knock-out CRISPR screens in mouse bone marrow–derived macrophages (BMDMs) [7] and in mouse RAW 264.7 cells [8], as well as for targeted knock-outs using Cas9-ribonucleoprotein complex nucleofection in mouse BMDMs [9]. A recent study reported a protocol for inserting transgenes into the Rosa26 locus of RAW 264.7 cells using CRISPR/Cas9 technology coupled to electroporation of plasmid DNA and growing up a bulk population of edited cells [10]. We developed the protocol that we report here to use CRISPR/Cas9 genome editing to create clonal populations of macrophages with knock-in fluorescent tags at diverse genomic loci in order to study stimulus-responsive gene expression at a single-cell level by imaging the fluorescent protein over time in single cells [11]. We base our CRISPR design on the strategy first developed by Ran and colleagues and use their pSpCas9 plasmid and one-step restriction-ligation cloning strategy for the guide RNA (gRNA) [12]. In this protocol, we use the Neon transfection system to deliver the gRNA, Cas9, and HDR donor sequences as circular plasmid DNA, coupled to a simple strategy for growing up clonal populations following a single single-cell sorting step. In this paper, we use a knock-in of YFP at the C-terminus of the IRF1 protein as our example, but we have used this approach for at least six different loci. This protocol works best for fluorescent proteins because FACS is used to sort positive cells, and positive cells are quite rare. However, it may be possible to adapt this protocol to non-fluorescent knock-ins in the future. At the end of this protocol, we also describe how we prepare an imaging experiment with the edited cells.

## Materials and reagents

**Biological materials**

1. RAW 264.7 mouse macrophage-like cells (ATCC, catalog number: TIB-71)
2. NIH3T3 mouse embryonic fibroblasts (ATCC, catalog number: CRL-1658)
3. pSp-Cas9(BB)-2A-Puro (PX459) plasmid (Addgene, catalog number: 62988)
4. pUC19 plasmid (Addgene, catalog number: 50005)

**Reagents**

1. Neon Transfection System 100 μL kit (Thermo Fisher, catalog number: MPK10025)
2. HyClone^TM^ classical liquid media, Dulbecco’s modified Eagles medium, high glucose, GE healthcare cell culture (DMEM)/high: with 4500 mg/L glucose and 4.0 mM L-Glutamine, without sodium pyruvate (VWR, catalog number: 16750-072)
3. Fetal bovine serum (FBS) (Fisher, catalog number: MT35010CV)
4. DPBS, no calcium, no magnesium (Thermo Fisher, catalog number: 14190250)
5. Penicillin-Streptomycin solution, 100×, 10,000 IU penicillin, 10,000 μg/mL streptomycin (Fisher, catalog number: MT30002CI)
6. Gibco DMEM, high glucose, no glutamine, no phenol red (Thermo Fisher, catalog number: 31053036)
7. Gibco L-Glutamine (200 mM) (Thermo Fisher, catalog number: 25030081)
8. T4 DNA ligase reaction buffer (NEB, catalog number: B0202S)
9. T4 polynucleotide kinase (PNK) (NEB, catalog number: M0201S)
10. 10× Tango buffer (Thermo Fisher, catalog number: BY5)
11. Dithiothreitol (DTT) (Millipore Sigma, catalog number: D9799-5g)
12. 10 mM ATP (NEB, catalog number: P0756S)
13. Bbs1-HF (NEB, catalog number: R3539S)
14. T7 DNA ligase (enzymatics, catalog number: L6020L)
15. Autoclaved MilliQ water
16. NEB 5-alpha competent *E. coli* (high efficiency) (NEB, catalog number: C2987I)
17. Opti-MEM reduced serum medium (Thermo Fisher, catalog number: 31985070)
18. Lipofectamine 2000 transfection reagent (Thermo Fisher, catalog number: 11668027)
19. Puromycin dihydrochloride (Millipore Sigma, catalog number: P8833-25MG)
20. Macherey-Nagel Nucleobond Xtra Midi Plus EF kit (Macherey Nagel, catalog number: 740422.5)
21. QIAprep Spin Miniprep kit (Qiagen, catalog number: 27104)
22. Zymo Quick-gDNA MiniPrep kit capped columns (Zymo Research, catalog number: D3024)
23. QIAquick Gel Extraction kit (Qiagen, catalog number: 28704)
24. Zymo DNA Clean & Concentrator-5 kit capped columns (Zymo Research, catalog number: D4013)
25. Phusion High-Fidelity DNA Polymerase-100u (NEB, catalog number: M0530S)
26. NEBuilder HiFi DNA Assembly Master Mix (NEB, catalog number: E2621S)
27. EDTA powder (Sigma, catalog number: E9884)
28. HEPES 1 M (Gibco, catalog number: 15630080)
29. Luria Broth (LB)-carbenicillin agar plates, with carbenicillin at 100 μg/mL
30. Luria Broth (LB)
31. Carbenicillin (Fisher BioReagents, catalog number: BP26485), stock at 100 mg/mL
32. Corning 0.25% Trypsin, 0.1% EDTA in HBSS w/o calcium, magnesium, and sodium bicarbonate; 6/PK 25-053-CI (Fisher, catalog number: MT25053CI)

**Solutions**

1. Complete DMEM (See Recipes)
2. Conditioned DMEM (See Recipes)
3. Antibiotic-free DMEM (See Recipes)
4. 0.1 M EDTA solution (See Recipes)
5. FACS sorting buffer (See Recipes)
6. Phenol Red–free DMEM (See Recipes)
7. 1,000× Puromycin solution (See Recipes)

**Recipes**

1. **Complete DMEM**
  **Table:** 

Reagent	Final concentration	Quantity or Volume
HyClone^TM^ DMEM	N/A	445 mL
FBS	10%	50 mL
Penicillin-Streptomycin solution, 100×	1%	5 mL
Total	n/a	500 mL
  Combine all ingredients and pass through a 0.2 μm filter bottle (Corning).
2. **Conditioned DMEM**
  **Table:** 

Reagent	Final concentration	Quantity or Volume
Supernatant from RAW 264.7 culture (RAW 264.7 cells split from a confluent plate at 1:8 density in complete DMEM with 10% FBS, 1% pen/strep, supernatant is collected after 24 h)	50%	100 mL
HyClone^TM^ DMEM	n/a	69 mL
FBS	20%	30 mL
Penicillin-Streptomycin solution, 100x	1%	1 mL
Total	n/a	200 mL
  Combine all ingredients and pass through a 0.2 μm filter bottle (Corning).
3. **Antibiotic-free DMEM**
  **Table:** 

Reagent	Final concentration	Quantity or Volume
HyClone^TM^ DMEM	n/a	450 mL
FBS	10%	50 mL
Total	n/a	500 mL
  Combine all ingredients and pass through a 0.2 μm filter bottle (Corning).
4. **0.1 M EDTA**
  **Table:** 

Reagent	Final concentration	Quantity or Volume
Autoclaved MilliQ water	n/a	Make up to 50 mL
EDTA powder	0.1 M	1.46 g
Total	n/a	50 mL
  To make the 0.1 M EDTA, dissolve the EDTA powder in 45 mL of autoclaved MilliQ water, then top off to 50 mL once totally dissolved for a total volume of 50 mL. Filter this solution through a 0.2 μm syringe filter (Acrodisc) before use.
5. **FACS sorting buffer**
  **Table:** 

Reagent	Final concentration	Quantity or Volume
DPBS	n/a	47.75 mL
EDTA 0.1 M (from Recipe 4)	1 mM	500 µL
HEPES 1 M	25 mM	1.25 mL
FBS	1 %	500 µL
Total	n/a	50 mL
  To make the FACS sorting buffer, combine all reagents and then pass through a 0.2 μm syringe filter (Acrodisc).
*Note: Other compositions of FACS sorting buffer will likely also work.*
6. **Phenol Red–free DMEM**
  **Table:** 

Reagent	Final concentration	Quantity or Volume
Gibco DMEM, no phenol red	N/A	440 mL
FBS	10%	50 mL
Penicillin-Streptomycin solution, 100×	1%	5 mL
L-Glutamine (200 mM)	2 mM	5 mL
Total	n/a	500 mL
  Combine all ingredients and pass through a 0.2 μm filter bottle (Corning).
7. **1,000× Puromycin solution (1 mg/mL)**
  **Table:** 

Reagent	Final concentration	Quantity or Volume
Autoclaved MilliQ water	n/a	Make up to 10 mL
Puromycin dihydrochloride	1 mg/mL	10 mg
Total	n/a	10 mL
  To make the 1,000**×** puromycin, dissolve the puromycin dihydrochloride in 9 mL of autoclaved MilliQ water, then top off to 10 mL once totally dissolved for a total volume of 10 mL. Filter this solution through a 0.2 μm syringe filter (Acrodisc) before use and store at -20 °C in 1 mL aliquots.

**Laboratory supplies**

1. Falcon 5 mL round bottom polystyrene test tube, with cell strainer snap cap (Falcon, catalog number: 352235)
2. GenClone 96-well cell culture plates flat bottom wells, TC treated (Genesee, catalog number: 25-109)
3. Falcon tissue culture plates 24 well (Fisher, catalog number: 087721H)
4. GenClone 6-well TC treated plates (Genesee, catalog number: 25-105MP)
5. GenClone TC treated dishes, 100 × 20 mm vented (Genesee, catalog number: 25-202)
6. Acrodisc syringe filter 0.2 µm Supor membrane, low protein binding, non-pyrogenic, PN 4612 (VWR, catalog number: 28143-310)
7. Pipette tips, serological pipettes, Eppendorf tubes, PCR tubes, 50 mL conical tubes, 15 mL conical tubes, 50 mL syringes
8. Cell scraper (e.g., VWR, catalog number: 75799-934)
9. Corning^®^ 500 mL vacuum filter/storage bottle system, 0.22 µm pore 33.2 cm PES membrane, sterile (Corning, catalog number: 431097)

## Equipment

1. Neon transfection system (Thermo Fisher, model: MPK5000)
2. PCR thermocycler (e.g., Thermo Fisher, model: ProFlex PCR System Thermocycler)
3. Hemacytometer (e.g., Hausser Scientific Hemacytometer Thermo Fisher, model: S17040)
4. Agarose gel electrophoresis rigs
5. Fluorescence microscope (e.g., Nikon, model: Eclipse Ti)
6. FACS machine (e.g., BD, model: FACSAria Fusion)
7. Benchtop mini centrifuge (e.g., MyFuge, model: Southern Labware C1012)
8. Benchtop microcentrifuge (e.g., Eppendorf, model: 5420)

## Software and datasets

1. ApE (plasmid editor) v3.1.3 November 11, 2022. Free, but you can equally well use a paid cloning software such as SnapGene if you prefer

## Procedure

- **Guide RNA (gRNA) design and testing (following the procedure from Ran et al. [12]) (Figure 1)**
  **Figure 1.**
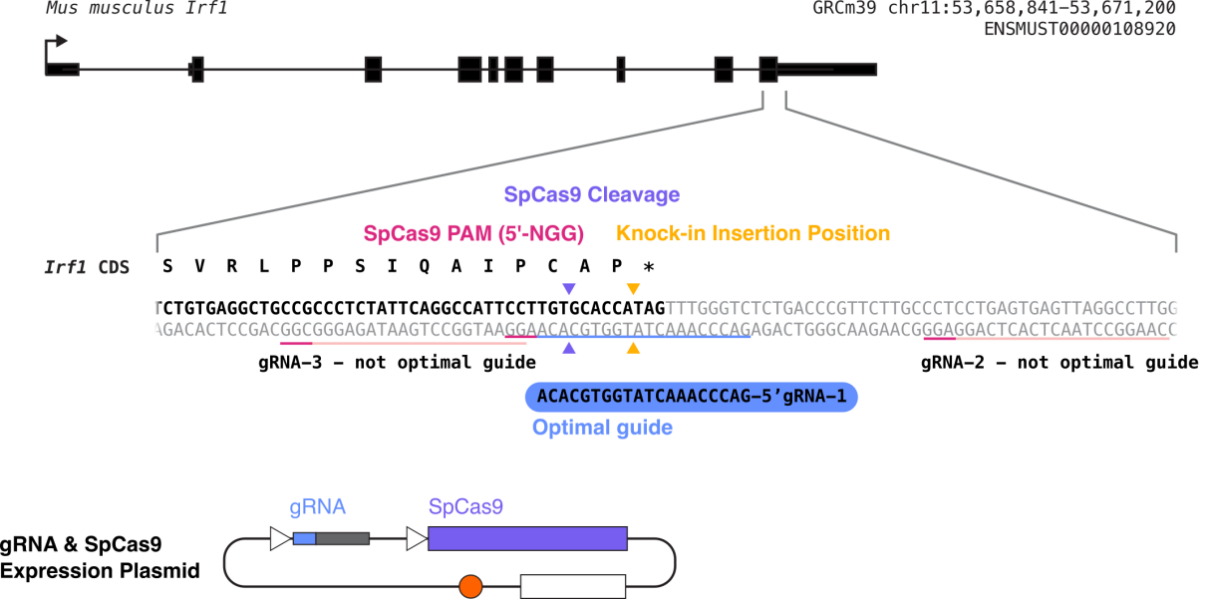
Diagram summarizing what will be accomplished in step A of this procedure. We will design multiple gRNAs, in this case targeting the C-terminal end of mouse IRF1, will clone each of them into a gRNA-Cas9 expression plasmid, and will test them to determine the optimal guide RNA (in this example, gRNA 1). The light pink or blue underlines in the sequence indicate the gRNA sequences, and the dark pink underlines indicate the PAM sequences for each possible gRNA. The desired insertion site is marked with yellow arrows, and the cleavage site for gRNA 1 is marked with purple arrows.
  1. Design guide RNAs
    - Identify where in the mouse genome you want to insert your knock-in sequence. For endogenous gene tagging, this should either be immediately after the endogenous ATG or immediately before the endogenous stop codon.
    - Select the genomic DNA sequence from 50 base pairs before to 50 base pairs after your desired insertion site.
    - Copy this sequence into crispor.tefor.net ( http://crispor.tefor.net/) to design guides. For the genome, choose *Mus musculus* mm10 or mm39. For selecting a PAM, select “20bp-NGG – Sp Cas9, SpCas9-HF1, eSpCas9 1.1.”
    - Select guides with high specificity scores and as high as possible cutting efficiency scores. You should additionally optimize for being as close to your desired insertion site as possible. You should select the three most promising guides (Figure 2).
*Note: Rarely do you have guides that look perfect here. This is part of why we screen three guides, so that you can choose several that look promising and then test them. The cutting efficiency prediction algorithms are not accurate for all cell types or loci, and this is also why we test the guides.*
      **Figure 2.**
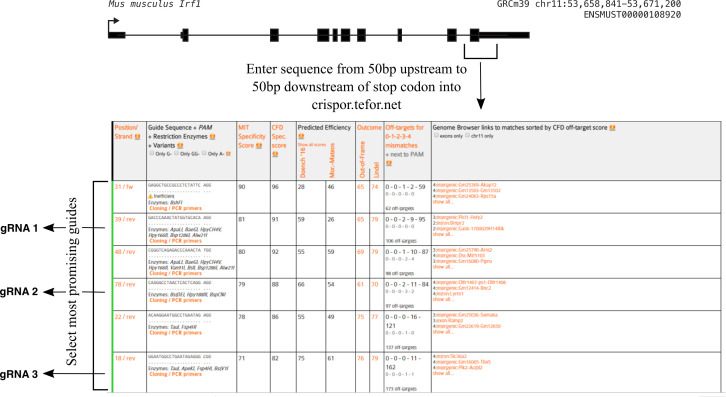
Example output from crispor.tefor.net ( http://crispor.tefor.net/ ). Select the 100 bp region from 50 bp upstream to 50 bp downstream of your desired knock-in insertion position, enter it into cirspor.tefor.net, and then analyze the possible gRNA results to choose the three most promising guides based on having a high predicted efficiency, high specificity score, and being as close to your desired insertion site as possible. In this example, gRNAs 1, 2, and 3 best meet these criteria.
    - Order two oligos (one forward and one reverse) per guide (from Eurofins, IDT, etc.). For the forward oligo, append CAACG to the 5’ end of the guide sequence. For the reverse oligo, take the reverse-complement of the forward guide sequence (not including the added CAACG) and add AAAC to the 5’ end and C to the 3’ end. For example, if your guide sequence was ACCCTT…GGGCT, then you would order forward oligo caacgACCCTT….GGGCT and reverse oligo aaacAGCCC…AAGGGTc.
  2. Clone the gRNA oligos into the pSpCas9-puro plasmid
    - Anneal oligos.
      i. Dilute oligos to 100 µM each in autoclaved MilliQ water based on instructions from the manufacturer.
      ii. In a PCR tube, combine 1 µL of each gRNA oligo (forward and reverse), 1 µL of T4 DNA ligase reaction buffer, 1 µL of T4 PNK, and 6 µL of autoclaved MilliQ water. Flick the tube and spin it down in a benchtop mini centrifuge.
      iii. Put this tube in the thermocycler with a cycle of 37 °C for 30 min, 95 °C for 5 min, ramp down to 25 °C at 0.1 °C/s, hold 1 min at 25 °C, hold at 4 °C. This takes approximately 1 h to run.
      iv. **Pause point:** Annealed oligos can be frozen at -20 °C for at least a year before use.
    - Clone the annealed gRNA oligos into the pSpCas9(BB)-2A-puro plasmid using the standard one-step restriction-ligation protocol described in Ran et al. [12].
      i. Dilute the annealed oligo 1:200 by adding 1 µL of annealed oligo (from above) to 199 µL of autoclaved MilliQ water.
      ii. Prepare the gRNA-Cas9 cloning reaction in a PCR tube on ice. Once the reagents are combined, flick the tube with your fingernail and spin it down in a benchtop mini centrifuge.
      **Table:** 

Component	Volume (µL)
pSpCas9(BB)-2A-puro plasmid (100 ng/µL)	1
1:200 diluted oligo	2
10× Tango buffer	2
DTT (10 mM)	1
ATP (10 mM)	1
Bbs1-HF	0.5
T7 DNA ligase	0.5
Autoclaved MilliQ water	12
Total	20
      iii. Incubate this tube in a thermocycler for six cycles of (37 °C for 5 min followed by 21 °C for 5 min) and then hold at 4 °C. This cycle takes approximately 1 h.
      iv. **Pause point:** After removal from the thermocycler, this can be stored at -20 °C for at least a year.
    - Transform 5 µL of this gRNA-Cas9 reaction into 50 μL of NEB 5-alpha competent *E. coli* (high efficiency).
      i. Place competent cells on ice for 10 min.
      ii. Add 5 µL of gRNA-Cas9 reaction to the cells and mix gently by tapping the tube.
      iii. Incubate cells + DNA on ice for 15–30 min.
      iv. Heat shock the cells + DNA tube for 30 s at 42 °C.
      v. Incubate the tube for 2 min on ice.
      vi. Add 200 μL of LB to the tube.
      vii. Shake at 300 rpm for 30–60 min at 37 °C.
      viii. Plate on LB-carbenicillin agar plate.
      ix. Place LB-carbenicillin plate in a 37 °C incubator overnight.
*Note: Other types of competent cells will likely also work.*
    - Pick three colonies from the plate that has grown up overnight and grow up each picked colony in a 5 mL culture of LB + carbenicillin. Perform minipreps using the QIAprep Spin Miniprep kit to extract the plasmids from the bacteria and sequence the plasmids using a universal U6 primer to confirm correct gRNA insertion. Re-streak the minipreps with the correct plasmid sequence onto new LB-carbenicillin plates and grow up overnight.
    - Pick a colony from the re-streak plate that had the correct sequencing and grow up this colony in a midiprep culture of 100 mL of LB + carbenicillin (so adding 100 μL of 1,000× carbenicillin). The culture should be grown overnight at 37 °C with shaking at 300 rpm.
    - Use the Macherey Nagel NucleoBond Midi Plus EF kit to perform a midiprep to extract the plasmid from the midiprep culture. Follow the kit instructions for midipreps of low-copy plasmids. For clarification and loading, we load the entire mix onto the filter (rather than spinning it out first); for DNA concentration at the end, we use the finalizers. We elute in 600 µL of provided water. This resulting plasmid is the pSp-Cas9-puro gRNA plasmid that you will use in subsequent steps.
*Note: Other midiprep kits could likely be used, but it is important for the final DNA concentration to be high, ideally over 1 μg/μL. For transfection later into macrophages, it is also important for the DNA to be endotoxin-free, which this kit accomplishes.*
  3. Test gRNA cutting efficiency
    - On day 1, use your hemocytometer to count the cells and seed 2 × 10^5^ NIH3T3 cells per well in a 6-well plate in complete DMEM. Seed the same number of wells as gRNAs that you are testing (usually three) plus one well as a control for uncut genomic DNA and one well as a control for puromycin selection.
*Note: We use NIH3T3 cells to test the gRNA cutting efficiency because they can be transiently transfected with a high efficiency and therefore selected for transfected cells while the cells are still expressing the transient selectable marker. RAW 264.7 cells transfected using the Neon system are too sick immediately after transfection to be selected using puromycin. We find that gRNAs selected for high efficiency in NIH3T3 cells are very effective for creating knock-ins in RAW 264.7 cells.*
    - The next day (day 2), for each gRNA to test, transfect one well.
      i. Add 2.5 μg of pSpCas9-puro gRNA plasmid DNA to 125 μL of Opti-MEM in an Eppendorf tube.
      ii. Add 7.5 μL (for a 1:3 ratio with the DNA) of Lipofectamine 2000 transfection reagent to 125 μL of Opti-MEM in a second Eppendorf tube.
      iii. Add the Lipofectamine + Opti-MEM solution to the DNA + Opti-MEM solution, pipette up and down gently, and incubate for 5 min at room temperature.
*Note: Be gentle with this solution, as the DNA–lipofectamine complex can be fragile.*
      iv. Add the transfection solution dropwise onto the well, gently tilt the plate to mix, and return the plate to the incubator.
    - On day 3, trypsinize the wells and transfer the entire contents of each well into its own 10 cm plate in complete DMEM. For the gRNA test plates and the puromycin control plate, add 10 μL of 1 mg/mL puromycin in 10 mL of total media.
    - On day 5, inspect the puromycin control plate and confirm that all cells are dead. Wash the gRNA test plates with DPBS and add fresh complete DMEM (without puromycin) onto the plate. Split the uncut genomic DNA control plate to be confluent the next day (usually 1:3).
    - On day 6, collect the cells using trypsin (each condition of gRNA test cells and the uncut control), spin down into cell pellets, and store at -80 °C.
  4. Extract genomic DNA from cell pellets
    - We use the Zymo Quick-gDNA Miniprep kit to extract gDNA; whichever gDNA extraction kit you prefer should work.
  5. Amplify the region where the cut should occur
    - Design PCR primers to amplify from ~300–600 bp upstream of the desired cut to ~300–600 bp downstream of the desired cut.
*Note: We use PrimerBlast (* *https://www.ncbi.nlm.nih.gov/tools/primer-blast/* *) from NCBI to identify primer pairs, aiming for 20 bp primers and an annealing temperature of 60 °C.*
    - Use these primers to amplify this region using PCR from the DNA you extracted from the gRNA test cells as well as the control uncut cells.
*Note: We use the NEB Phusion DNA polymerase and associated reagents, but alternative polymerase systems will likely also work. As the Phusion polymerase requires a different Tm for primers than is generally calculated, we use the NEB Tm calculator (https://tmcalculator.neb.com/#!/main) to calculate the Tm we will use in the PCR program. We use a thermocycler program of 98 °C for 30 s, then 30 cycles of (98 °C for 10 s, annealing temp for 30 s, 72 °C for 1 min per kilobase of PCR product), then 72 °C for 10 min, hold at 4 °C.*
    - Run the PCR product on a 1% agarose gel to confirm band size.
    - Gel extract the PCR product from the gel using the QIAquick Gel Extraction kit.
*Note: Any gel extraction kit should work.*
    - Send the uncut control and gRNA test amplicons that you gel extracted for Sanger sequencing with the primers you used for the PCR.
*Note: Occasionally, these primers will be bad sequencing primers; in this case, you can design and order an additional internal primer to sequence across the cut site.*
    - Use the online Synthego ICE tool ( https://ice.synthego.com/#/) to assess gRNA cutting efficiency.
*Note: This tool compares the .ab1 Sanger sequencing files in uncut (control) cells and cells transfected with a gRNA to determine the fraction of cells with an indel at the CRISPR site. The software also requires you to input the guide sequence used for that sample. The fraction of cells with an indel correlates with the gRNA cutting efficiency, which is also calculated by the software. The software also shows exactly which indels were formed (Figure 3).*
    - Choose the gRNA with the best cutting efficiency that is as close to your desired insertion site as possible.
*Note: We select the gRNA with the highest cutting efficiency (indel percentage) that is as close to our desired insertion site as possible. In our experience, any indel percentage above 50% is generally sufficient, though we have not specifically tested moving forward with a gRNA with a score of 40% vs. 60%, for example. We have seen gRNAs with an indel score below 30% fail, though we cannot necessarily attribute their failure specifically to their low cutting efficiency. In the IRF1 example, we chose gRNA 1.*
      **Figure 3.**
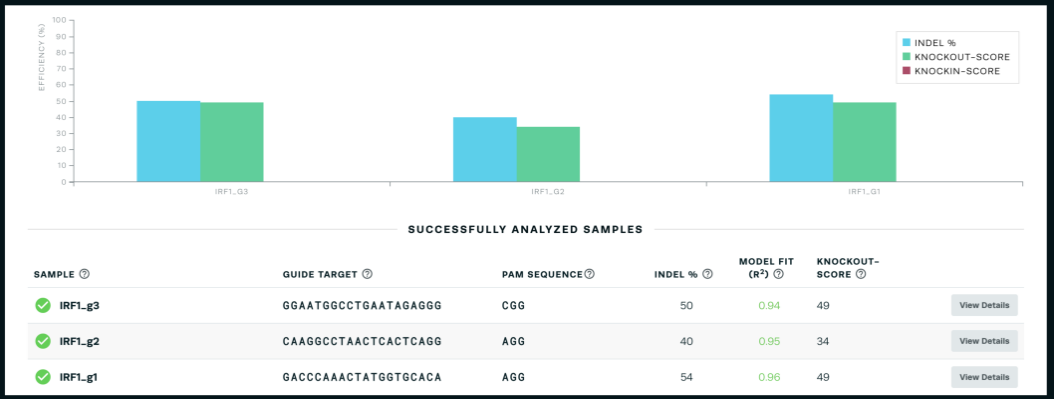
Sample output from the online Synthego ICE tool ( **https://ice.synthego.com/#/** ) **showing the indel percentage and knock-out score**
- **Design and build HDR donor plasmid**
  1. Use NEB Phusion polymerase to amplify ~1 kb homology arms on each side of the desired insertion site from RAW 264.7 genomic DNA. We use the Zymo Quick-gDNA Miniprep kit to extract RAW 264.7 genomic DNA from wild-type RAW 264.7 cells. For N-terminal tagging, the insertion site should be immediately after the endogenous ATG start codon and, for C-terminal tagging, it should be immediately before the endogenous stop codon. Once the best gRNA has been identified, its PAM sequence needs to be mutated in the donor plasmid to avoid re-cutting. We do this using primers with overhangs to add new sequence. If the PAM cannot be mutated synonymously, then at least three synonymous mutations should be made in the seed sequence of the gRNA so that Cas9 re-binding is prevented in that way. If the PAM is in a UTR, we do a literature search to identify if it is in a region of the UTR known to be important for regulation; if not, we simply mutate the PAM. If there is known regulation in the UTR, we try to avoid mutating that region. The desired fluorescent protein insertion sequence also needs to be amplified via PCR from a plasmid containing that fluorescent protein. We assemble the plasmids using Gibson assembly in a pUC19 backbone and so add the appropriate Gibson overhangs on our primers as well when needed. This generally results in a four-part Gibson assembly reaction with the plasmid backbone, left homology arm, right homology arm, and fluorescent protein (Figure 4).
    **Figure 4.**
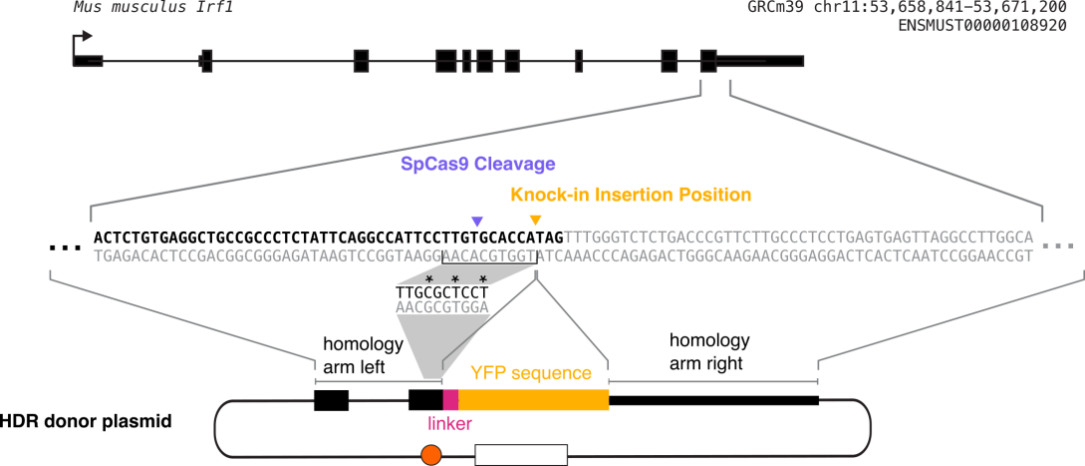
Diagram of construction of homology-directed repair (HDR) donor plasmid. The plasmid consists of the left homology arm, linker sequence, fluorescent protein tag, and right homology arm. Each of these sequences must be PCR-amplified out of either the genomic DNA (for the homology arms) or a fluorescent protein plasmid (for the fluorescent protein). As this is a C-terminal tag, the fluorescent protein is inserted directly before the endogenous stop codon. The grey shading indicates the process of mutating the gRNA recognition sequence synonymously to prevent further gRNA binding without changing the protein sequence. The homology arm incorporated in the donor plasmid contains this mutated sequence, and the mutated bases are marked with asterisks in this diagram. Each homology arm should be ~1 kb long.
*Notes:*
    - *You can use a different PCR chemistry, but we recommend a high-fidelity PCR enzyme because it is important for the entire plasmid to have the correct sequence.*
    - *For N-terminal tagging, be sure to not include a stop codon on the fluorescent protein tag.*
    - *We use PrimerBlast (* *https://www.ncbi.nlm.nih.gov/tools/primer-blast/* *) to design our cloning primers, aiming for 20 bp primers and an annealing temperature of 60 °C.*
    - *We use restriction enzymes to digest 2 μg of the pUC19 backbone. We then run this digested backbone as well as our three PCR fragments (left homology arm, right homology arm, and fluorescent protein tag) on an agarose gel, gel extract the bands using the QIAquick gel extraction kit where we elute in 30 µL, and then run that product through a Zymo DNA Clean & Concentrator-5 kit and elute in 12 μL of the Zymo elution buffer. This two-step purification results in cleaner, higher-concentration DNA that leads to better efficiency with Gibson assembly in our hands. We do our Gibson assembly reactions using 100 ng of each DNA piece and use the NEBuilder HiFi DNA Assembly Master Mix, incubating for 1 h at 50 °C. We design our Gibson cloning to have ~23 bp overlaps between each two DNA pieces.*
    - *If you are adding linker or T2A/P2A sequences into the plasmids between the endogenous protein and the fluorescent tag, these can also be introduced on primer tails when designing your PCR. We use the T2A sequence from Nora et al. [6] and a GDGAGLIN linker with DNA sequence GGCGACGGCGCCGGCCTGATCAAC.*
  2. Transform the cloned plasmid (after Gibson assembly) into NEB 5-alpha competent *E. coli* (high efficiency) as above.
  3. Grow up four minipreps, sequence the minipreps, and identify a miniprep with the correct sequencing. You want to be sure the entire homology arm and insertion sequences are correct.
  4. Grow up a midiprep culture of the plasmid with the correct sequencing and perform the midiprep using the Macherey Nagel NucleoBond Midi Plus EF kit in the same way as described for the gRNA-Cas9 plasmid above.
*Note: Plasmid concentration is best if above 1 μg/μL. If it is below 500 ng/μL, we suggest repeating the midiprep.*
- **Transfect gRNA-Cas9 plasmid and donor plasmid into RAW 264.7 cells (Figure 5)**
*Note: We use the Neon transfection system rather than any form of lipofection because, in our hands, the Neon transfection system gives much higher transfection efficiency than any lipofection method in RAW 264.7 cells. See General Note 6.*
  1. Have a confluent 10 cm plate of RAW 264.7 cells ready on the day of transfection.
  2. Fill three wells of a 6-well plate with 2 mL each of antibiotic-free DMEM.
*Note: It is important to use the antibiotic-free DMEM here, as having antibiotics in the media decreases cell viability after transfection.*
  3. In an Eppendorf tube, combine 45 μg of gRNA-Cas9 plasmid and 45 μg of donor plasmid. Ideally, both the donor and gRNA-Cas9 plasmid concentrations are over 1 μg/μL.
    - In this protocol, we transfect in triplicate, so each single transfection is 15 μg of each plasmid.
*Note: We attempted various ratios of gRNA-Cas9 DNA to donor DNA, different volumes of DNA, pretreating the cells with DMSO, pretreating with interferon-gamma (as our genes were interferon-gamma inducible and the idea was to open up the chromatin at their loci), and also plating post-transfection into media containing 2 μM M3814 (M3814 is a DNA-PK inhibitor that can increase the ratio of HDR edits to NHEJ edits [13]). Adding more DNA decreased the cell viability after transfection, and we saw little effect of either DMSO pretreatment or M3814 post-treatment. Therefore, we chose not to either pretreat or post-treat our cells for our experiments.*
*Note: We do not linearize our plasmids before transfection, but we did not test how linearization might affect transfection or knock-in efficiency. One reason we did not attempt plasmid linearization was concern over the possible introduction of endotoxins in this process. Therefore, we are transfecting circular plasmid DNA.*
    **Figure 5.**
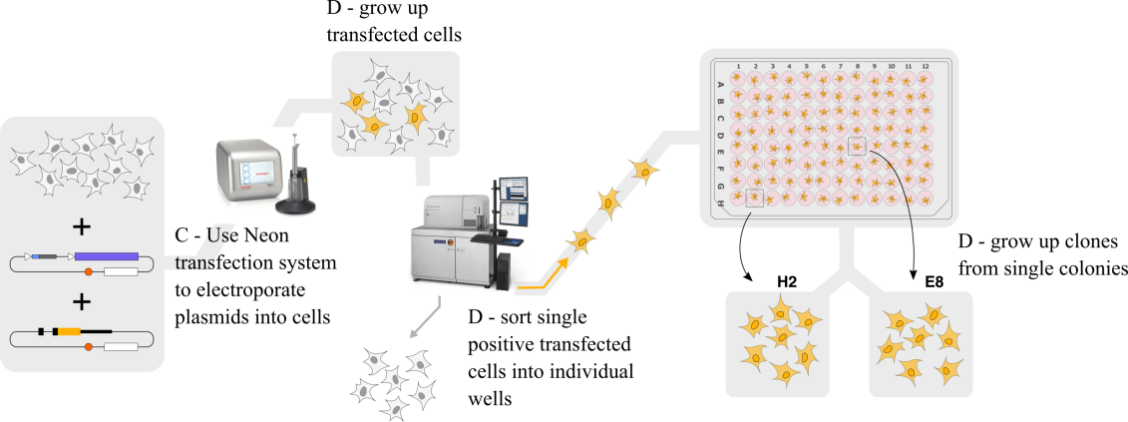
Graphical outline of steps C and D. We transfect the gRNA-Cas9 and homology-directed repair (HDR) donor plasmids into the cells, grow up transfected cells, sort single positive transfected cells into individual wells using FACS, and grow up clones for further screening.
  4. Set up the Neon transfection system machine, program it for 1,680 V, 20 ms, 1 pulse, and set up the Neon cuvette with 3 mL of buffer E2 from the Neon electroporation reagent kit.
  5. Prepare an Eppendorf tube with 1 mL of DPBS for washing the Neon tip.
  6. Spin down 7.5 × 10^6^ RAW 264.7 cells at 200× *g* for 3 min and aspirate the supernatant.
    - In this protocol, we transfect in triplicate, so each single transfection is 2.5 × 10^6^ cells.
  7. Resuspend cells in 1 mL of DPBS, spin down again, and aspirate the supernatant.
  8. Resuspend the cells in 300 μL of R buffer from the Neon electroporation reagent kit.
*Note: The kit says that extended R buffer exposure can be harmful for cells, so move quickly after this step.*
  9. Add the cells in R buffer to the tube with your plasmid DNA and pipette up and down gently 3–5 times.
  10. Use a 100 μL tip on the Neon pipette to pick up 100 μL of the cells–buffer–DNA solution. Make sure there are no bubbles in the tip.
  11. Put the tip in the Neon cuvette and push down until it clicks.
  12. Press *Start* on the program (1,680 V, 20 ms, 1 pulse).
  13. Once the program has completed, gently eject the contents of the tip into one well of a 6-well plate with antibiotic-free DMEM.
  14. Insert the pipette tip into the tube of DPBS and pipette the DPBS up and down five times to wash.
  15. Repeat steps C10–14 twice more, for a total of three transfections, plating into a new well for each transfection.
  16. Once all transfections are complete, rock the cell plate back and forth to evenly distribute cells.
  17. Place the cell plate in your tissue culture incubator.
  18. Put away the Neon machine. According to Thermo Fisher, the tips are not reusable, and the cuvettes should only be reused a few times. In our experience, tips can be reused at least five times, but we use a new tip for each new cell line that we construct to avoid contamination. To store tips, we place them in a 15 mL conical tube. At least 24 h before reuse, we use forceps to separate the internal gold plunger from the external plastic, put both the plunger and plastic back in the 15 mL conical tube, and add filtered 100% ethanol for at least 2 h. We then dry them by placing them on a Kimwipe in a tissue culture hood with the UV light on for 1 h. We also reuse cuvettes at least five times, though we do sometimes see a decrease in cell viability after transfection using older cuvettes. We store cuvettes in a 50 mL conical tube with filtered 100% ethanol, and before reuse we remove them from the conical tube and dry them by placing them on a Kimwipe in a tissue culture hood with the UV light on for 1 h.
- **Grow up cells, sort single positive cells, and grow up clonal colonies from single cells**
  1. Two days after electroporation, inspect cell viability. There may be many floating cells, many of which are dead but some of which may be alive.
*Note: Cell viability after transfection can vary greatly based on a number of factors. It can depend on cell health before electroporation, age of reagents (buffers, cuvettes, etc.), as well as the nature and purity of the DNA. We have done electroporations of the same DNA into two different RAW 264.7 lines in parallel on the same day and had very different post-transfection viability between them. We have also electroporated different DNAs into the same cell line on the same day and had different viability. At two days post-transfection, an electroporation that had good viability will look like a ~50% confluent well of cells, but the cells are more elongated than they normally are, so 50% confluence here is fewer cells than 50% confluence of normally growing RAW 264.7 cells. An electroporation that had poor viability will look like sparse single cells on the bottom. However, we have still seen successful genome editing in cases of poor viability after transfection.*
  2. Use a cell scraper followed by strongly pipetting media against the bottom of the well to collect all cells in all three wells. Combine these into one 15 mL conical tube, spin down the conical tube at 200× *g* for 3 min, and aspirate the supernatant.
  3. Resuspend the cells in 2 mL of complete DMEM and place this suspension in a new well of a 6-well plate (so you have now combined three wells into one well).
  4. Monitor the well until it reaches confluence; this generally takes 2–5 days depending on cell viability.
  5. Expand these cells into a confluent 10 cm plate.
  6. We like to first inspect the cells under a microscope to see if they express the knocked-in fluorescent protein before sorting them. To do this, we plate two wells of a 6-well plate with these bulk transfected cells and image 60 positions per well every hour for 24 h, adding our stimulus after 2 h for stimulus-inducible genes. If you see the fluorescence expression, that is great! If not, sometimes the actual knock-ins are so rare that you can only pick up on them via FACS; we have had cases where we do not see fluorescence via microscopy but do pick up correctly edited cells via FACS. Be sure to only image a fraction of your bulk transfected population and keep most of it in the incubator to prepare for sorting (Figure 6).
  7. Sort single positive cells into a 96-well plate.
*Note: We generally sort 7–9 days after transfection. Occasionally, it will take longer for the cells to grow up sufficiently; that is ok too.*
    - Induce a 10 cm plate of transfected cells with whatever stimulus should express your fluorescent knock-in. If it is constitutively expressed, then skip this step.
    - Prepare two 96-well plates with 100 μL of conditioned DMEM per well.
*Note: Using conditioned rather than regular DMEM results in the single cells growing into colonies faster after sorting as well as a higher fraction of sorted single cells surviving and growing into colonies. This is a key step in this protocol.*
    - Collect your induced transfected cells, as well as the cells you made them from as a control for FACS gating.
    - Spin cells down at 200× *g* for 3 min, resuspend in ~3 mL of FACS sorting buffer, and pass through a filter into a FACS tube (Falcon 5 mL round bottom polystyrene test tube, with cell strainer snap cap).
    - Bring the FACS tubes with the cells on ice to a FACS machine. Gate for positive cells compared with the pre-transfection control. It is ok to get some false positives here because you will screen the clones further in subsequent steps. Sort cells that express your fluorescent protein higher than the control into the 96-well plates with one cell per well (Figure 7 ).
*Note: The fraction of positive cells is often very small, generally between 2% and 0.01% of the sample (see below for more discussion of this). However, this fraction is often enough to recover the knock-in cells.*
      **Figure 6.**
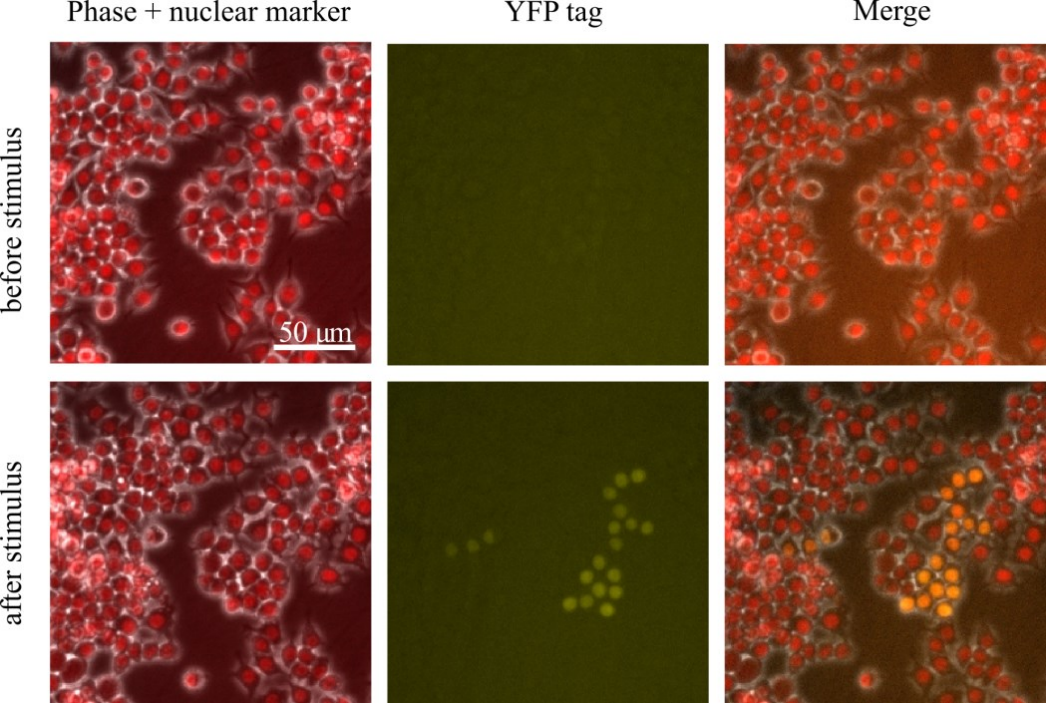
Sample field of view from imaging bulk transfected cells after they have grown to confluency where a gene has been tagged with YFP. In this field of view, there is a group of cells that express YFP after stimulus, which are the edited cells we are looking for. There are also many cells that do not express YFP.
      **Figure 7.**
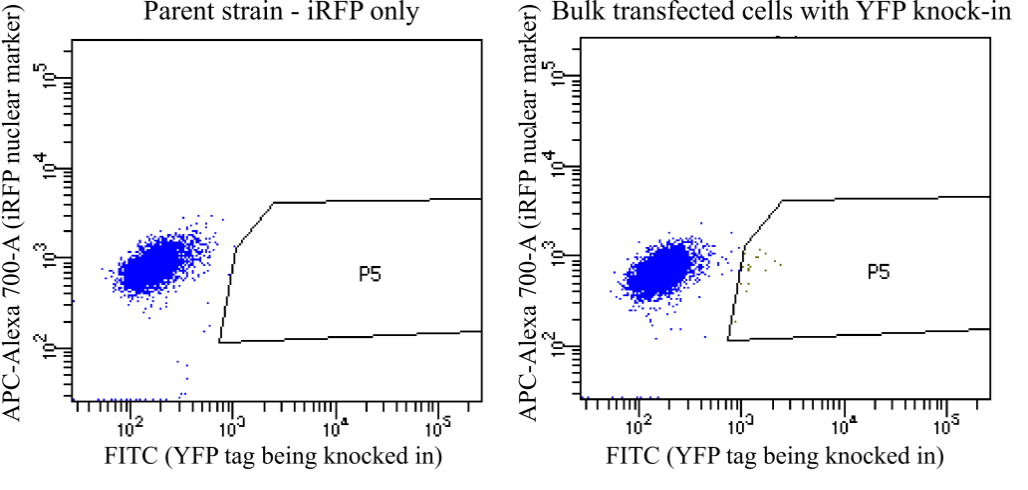
Sample FACS plots when sorting for positive cells. In this example, iRFP+ parent cells were transfected to knock in a YFP tag. These plots show YFP vs. iRFP for each cell being sorted. On the left are the parent cells, and on the right are the bulk transfected cells. P5 is the gate used to collect positive cells that have higher YFP than any cells in the parent population. In this example, in the bulk transfected cells, P5 is 0.3% of the alive, single-cell population and 0.2% of the total population. The true YFP-positive population likely extends to the left of the P5 gate shown here, but we drew the gate here to try to minimize false positives. In other situations, it may be worth it to take some false positives to avoid throwing out true positives and to screen out the false positives later when the clones are screened by microscopy.
  8. Grow up single colonies into clonal populations.
    - Seven to nine days after sorting, look at each plate under a regular tissue culture inverted microscope and identify wells with colonies growing in them.
*Note: We usually have between 30% and 60% of wells with growing colonies.*
    - Using a fluorescence microscope, image each growing colony in the channel where you expect to see your knock-in fluorescence.
    - If you are looking for stimulus-responsive expression, add stimulus, wait the appropriate time for your gene to turn on, and then image on the fluorescence microscope again.
    - Based on the fluorescence images, identify clones that are positive for fluorescence. We generally also screen by colony size and take the colonies on the bigger side. Choose 12 colonies to grow up (Figure 8).
*Note: Generally, if the editing worked, most (~70%–100%) clones that you screen will be positive for the fluorescence. If the editing did not work and you ended up sorting false positives, then no clones will be positive, or those that are will be positive even without induction for inducible genes.*
      **Figure 8.**
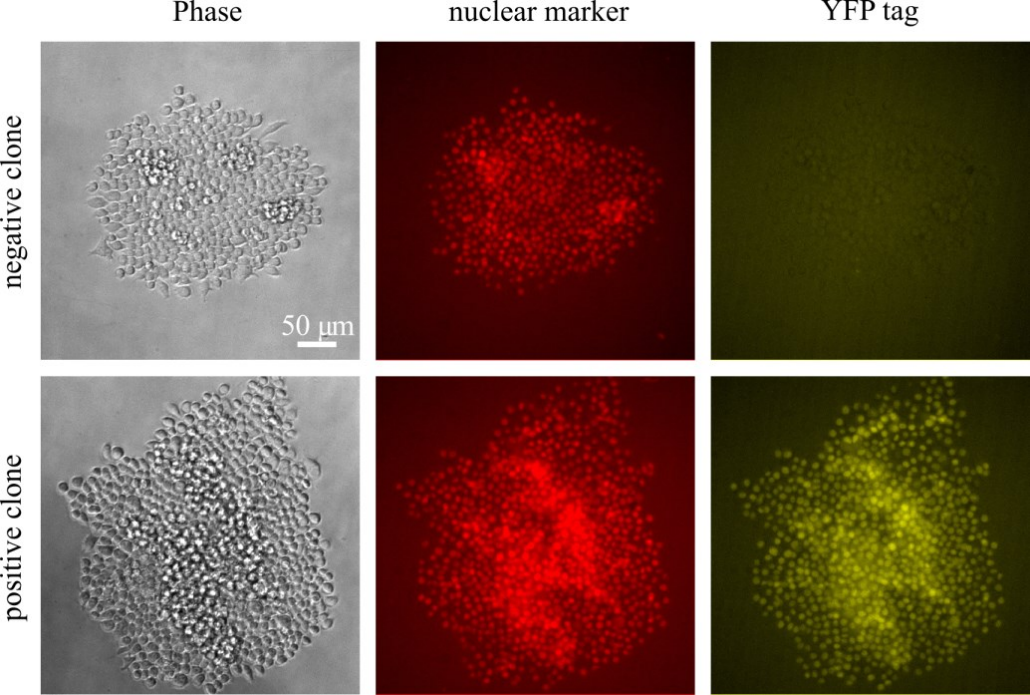
Sample images from screening clones in a 96-well plate eight days after sorting, showing both a positive clone and a negative clone for the knocked-in YFP tag in a nuclear marker (iRFP+) background
    - When the colony gets large in the 96-well plate (generally 10–12 days after sorting; it is ok to wait until the media turns yellow, but if you choose to wait that long, you should move the colony promptly after that), use forceful pipetting of media using a P200 pipette to dislodge the colony and move the cell suspension to a 24-well plate.
    - Continue to grow up and transfer the cells when confluent from a 24-well plate to a 6-well plate to a 10 cm plate.
- **Screen clones**
  1. For the 12 colonies that you grew up, spin down two cell pellets from a confluent 10 cm plate and store at -80 °C. Additionally, freeze cryovials of cells so that you can recover ones you want to use later.
  2. Extract the genomic DNA from the cell pellet for each clone; we use the Zymo Quick-gDNA Miniprep kit.
  3. Use PCR to amplify the region across the insertion site from the left into the right homology arm. We use the primers that we designed for determining gRNA cutting efficiency above. Use a long enough elongation time to elongate across the inserted DNA as well. We also run a control PCR on unedited wild-type DNA.
  4. Run this PCR product on a gel. Note the bands: if there are two bands, then the clone is likely heterozygous for the knock-in; if there is only one large band, it is likely homozygous for the knock-in; if there is only one small band, it likely does not have the knock-in (Figure 9).
    **Figure 9.**
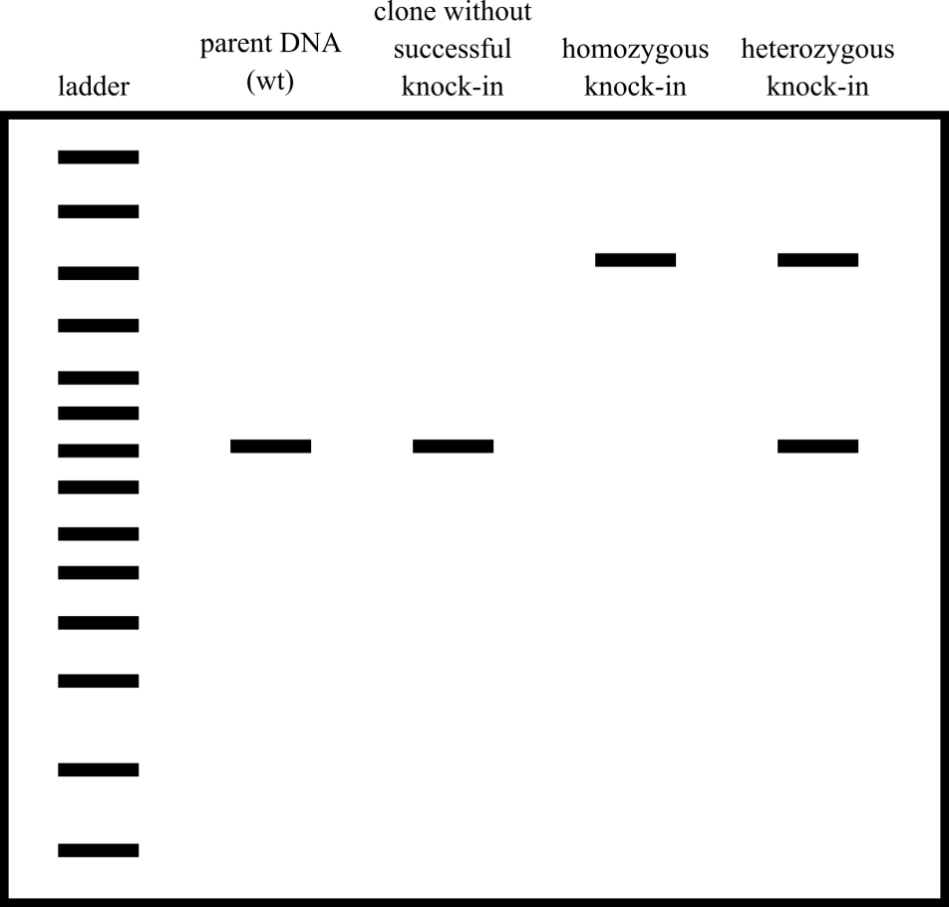
Cartoon example of a screening gel. Wild-type unedited cells have one small band, clones that do not have the knock-in inserted have one small band, homozygous knock-ins have one large band, and heterozygous knock-ins have one large band and one small band.
  5. Gel extract each band of DNA using the QIAquick gel extraction kit and send each band for sequencing to sequence the entire amplicon. We sequence with the same primers we used for the PCR.
  6. Analyze this sequencing data. For small bands, the goal is that the DNA sequence is identical to the uncut genomic DNA, rather than having been cut and repaired using non-homologous end joining to result in an indel. For large bands, the goal is to see precisely the endogenous sequence with your fluorescent protein inserted as you designed. In homozygous knock-ins, if the two alleles are slightly different, this will sometimes result in an .ab1 sequencing file with overlapping peaks as there are two sequences there. In this case, sequencing from the other side or an internal primer can help. It is also the case that you want any homozygous knock-ins to be identical, and so clones with overlapping-peak sequencing may not be the best choice and you may be able to discard them (Figure 10).
    **Figure 10.**
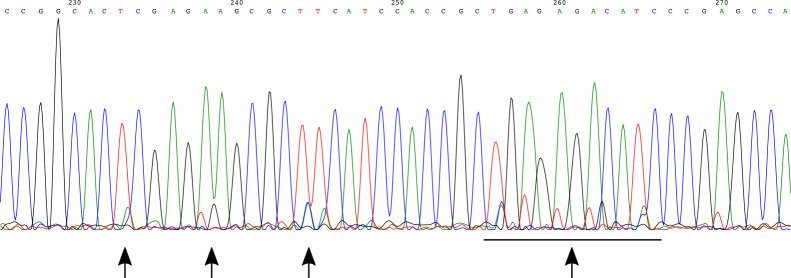
Example of a Sanger sequencing chromatogram with these *overlapping peaks.* This example is from tagging CXCL10 (instead of the IRF1 example used for the rest of this protocol), as we had no homozygous knock-ins in IRF1 and never saw this overlapping peak phenomenon there. Based on this chromatogram, we chose to not move forward with this particular clone. Arrows point to specific regions of overlapping peaks.
  7. Select the clones with the correct insertion sequences to move forward with further screening.
  8. For the rest of the screening, how you screen depends on what your tagged gene function is and what you are looking for. For inducible genes, we screen for fluorescence induction and choose a clone with representative fluorescence across all clones. We also do qPCR and Western blots where relevant to confirm near-endogenous RNA and protein expression level and timing for the tagged allele. If the tagged gene has a function (for example, as a transcription factor), we screen for gene function; for transcription factors, that means confirming the gene expression downstream of the transcription factor is at near-endogenous levels.
  9. Based on all these data, choose your preferred clone and work with that clone.
- **Set up an imaging experiment**
  1. The day before (ideally, ~20 h before) you wish to image, seed 4 × 10^4^ of your edited RAW 264.7 cells into each well of a 24-well plate in complete DMEM. We do this by preparing a tube with 104 × 10^4^ cells in 13 mL of media, mixing that tube well, and then adding 500 μL of that mix to each well. After adding the cells to all wells, shake the plate back and forth on the floor of the TC hood enough to really agitate the liquid but not enough for it to spill over between wells. This is important to ensure an even distribution of cells. Incubate the plate in a tissue culture incubator for ~20 h for the cells to settle on the plate.
*Note: This seeding density is dense enough for the cells to be happy, but relatively sparse so that the cells can sit for 20 h in the incubator and then be imaged for 48 h without starting to grow on top of each other. You may want to modify this density based on your imaging time.*
  2. Right before imaging, aspirate the media in each well and replace it with 500 μL of Phenol Red–free DMEM for imaging.
  3. Bring the plate to the microscope and set up on the microscope, including the temperature and CO_2_ incubation.
  4. Select exposure time and frequency; we take phase and iRFP images every 10 min for tracking and fluorescence in other channels every sixth cycle (every 60 min) to minimize phototoxicity.
  5. Select positions, ensuring that you are in the middle of each well (which should look like the part with the darkest background). We take two images per well for a total of 48 images; our microscope cannot take more images per well within a 10 min image frequency. We also move through the plate in an “S” pattern to avoid the stage moving the plate back and forth more than necessary.
  6. Run your experiment!

## Data analysis

As this protocol describes creation of cell lines, there is no data analysis.

## Validation of protocol

We have used this protocol to insert several different constructs into both the Tigre and Rosa26 safe harbor loci in RAW 264.7 cells. Additionally, we have used it to tag the genes IRF1, CXCL10, CXCL9, IRF8, and GBP1, all on the first try. We have tried and been unsuccessful in using this protocol (and its associated troubleshooting steps) to tag NOS2 and FCGR1, and when we tagged STAT1, its function was perturbed. A paper describing a cell line with EF1alpha-NLS-iRFP knocked into the Tigre locus and tags on IRF1, CXCL10, and CXCL9 is described in Fig 1A of Naigles 2023 and used extensively there [11].

## General notes and troubleshooting

**General notes**

1. There is a pSpCas9(BB)-2A-GFP plasmid that can be used in place of the pSpCas9(BB)-2A-puro plasmid. This would allow for visual assessment of cell transfection efficiency by the fraction of GFP-expressing cells two days after Neon transfection. This can be useful for troubleshooting if there is concern about low transfection efficiency. Depending on your experimental design, this may also be useful if you want to select for transfected cells at that time without using puromycin. Using the pSpCas9-GFP plasmid will not interfere with downstream screening (even of GFP insertions) because the transient expression of GFP will have ended by the 7–9 day timepoint when cells are screened by FACS in this protocol. However, a cell line that constitutively expresses GFP would not be a suitable background to use if you are trying to use the pSpCas9-GFP plasmid to assess transfection efficiency, as the constitutive GFP will mask the GFP transiently expressed by the Cas9-GFP construct. If you are knocking in a GFP construct without a functional promoter, you should have no issues with expression from your donor plasmid interfering with assessing transfection efficiency via screening for the GFP expression from the Cas9-GFP plasmid. However, if your donor plasmid expresses GFP and contains a promoter for the GFP, then at the 2–3 day post-transfection timepoint you will see expression from both the Cas9-GFP and donor GFP construct.
2. In this protocol, cells are not selected for successful transfection before being selected for successful knock-in of the fluorescent protein. This means that the fraction of the cells that are positive for successful knock-in is very low, as only a fraction of cells is successfully transfected and then only a fraction of those has successful CRISPR cutting and HDR repair. Our preliminary experiments showed that transfection efficiency using the Neon system as described here is approximately 40%–60%, but that the fraction of cells with the fluorescent protein knocked into the locus ranges from 2% to 0.01% and is most commonly around 0.4%–0.1%. This 2% to 0.01% range is high enough to obtain positive cells from FACS. It may be possible to use puromycin to select for transfected RAW 264.7 cells, as they will transiently express puromycin resistance. This approach would need to be optimized to not further decrease cell viability after transfection but could be worth trying if attempting to knock-in a non-fluorescent construct.
3. We did not try linearizing the donor DNA, so cannot comment on how that would affect HDR efficiency. We did not do this due to concerns over introduction of endotoxins and because the protocol worked sufficiently for our purposes without it.
4. In our experience, there are two genes that we have been unable to tag despite extensive efforts. While one of these genes is in a closed chromatin environment, we have successfully tagged other closed-chromatin genes. Pretreating the cells with DMSO or interferon-gamma to attempt to open the chromatin at this locus prior to transfection did not lead to successful tagging. One gene had the correct genomic sequence that would indicate successful tagging, but the fluorescence was too weak to image. This leads us to conclude that a certain minimal level of protein expression is needed for fluorescent tagging to be a useful strategy.
5. We attempted many variations on the Neon transfection described here, including varying the electroporation settings, quantity of cells, and quantity of DNA. The protocol described here works best for maximizing knock-in efficiency and minimizing cell death after electroporation. After we had settled on the electroporation setting and cell number per electroporation (2.5 × 10^6^), we tried pretreating the cells with DMSO for 24 h, pretreating with IFNγ for 2 h, increasing the donor DNA to be 30 μg rather than 15 μg, and plating the cells post-transfection into media containing 2 μM A3814, which inhibits DNA-dependent protein kinase (DNA-PK). DNA-PK plays a role in non-homologous end joining (NHEJ), and some studies have shown that its inhibition decreases NHEJ and so promotes HDR as the resolution pathway after double-strand breaks [13–15]. Overall, we found that pretreatment with IFNγ or using 30 µg of donor DNA decreased cell viability after transfection, while DMSO pretreatment did not. The DMSO pretreatment + 30 μg donor and the no pretreatment + 15 μg donor + plate into media with M3814 both had slightly higher knock-in rates than the no pretreatment + 15 μg donor case we present here. We chose to not use the higher quantity of donor DNA due to the cell toxicity, and later experiments showed less difference between plating into media with or without M3814, which also contributed to our choice to stop using the M3814. However, if there is a need to increase HDR efficiency specifically, then plating into media with M3814 is worth trying.
6. We tried a variety of other transfection methods for the RAW 264.7 cells, but in all cases had worse transfection efficiency than the Neon system. When using a constitutive fluorescent protein expression plasmid to test transfection efficiency, we got 5%–10% transfection efficiency with FuGene, up to 30% but more commonly 10% with TransIT-X2, 10% with polyethylenimine (PEI), 20% with Lipofectamine 2000, and <10% with GeneJet. For all these approaches, we tried a variety of conditions and DNA:reagent ratios, including those suggested by the supplier for RAW 264.7 cells. We used the Neon due to its much higher transfection efficiency (40%–60%); however, if another transient transfection approach works for RAW 264.7 cells in your hands, then it may also be usable for delivering plasmids for this endogenous tagging approach.

**Troubleshooting**

The main issue that occurs with this protocol is that you do the whole procedure and have no positive clones. In this situation, it is best to go back and make sure each previous step is working. Below are some of the intermediate steps that need to work to obtain the final knock-in. However, as discussed above, there are a couple of loci that we have been unable to tag using this protocol even after troubleshooting.

**Table:** 

Step	How to test	Possible ways to fix
gRNA-Cas9 cutting of DNA	Go back to the data where you test cutting efficiency in NIH3T3 cells; you can also do the same protocol to test in RAW 264.7 cells in case there are single nucleotide polymorphisms (SNPs) that lead to different efficiencies in each cell type.	Choose a different guide sequence.
Neon transfection	Confirm transfection efficiency using a plasmid that constitutively expresses a fluorescent protein.	Adjust quantity of cells or DNA, ensure cells are happy before transfection, minimize time in R buffer, minimize time cells are in a tube rather than a plate, try a new Neon tip or cuvette, replace Neon buffers.
HDR at the locus	There is no direct way to test for poor HDR because we only screen after both transfection and HDR, but if you have good cutting and transfection efficiency and still no knock-in cells, this may be due to a lack of HDR at the locus.	It is worth trying to pretreat with DMSO or a stimulus that opens the chromatin at your locus or to plate into media with M3814. It may also be worth trying to move to tag the other terminus if that is an option for your gene, as some termini just seem hard to tag for reasons we do not understand. You could also grow up some of the relatively brighter single-cell clones, amplify the locus using PCR, and analyze that sequencing, as it is possible that HDR was successful, but fluorescence intensity is just low.

Some additional issues that may occur are described below.

**Table:** 

Issue	Suggested solution
You only have heterozygous clones and want homozygous clones	Try to screen more clones. In our experience, most knock-ins are either all homozygous or all heterozygous, but we have had one case (in a safe harbor locus) where there has been a mix that can be seen by screening more clones.
All the intermediate steps work and you still have no clones	There are a number of reasons this might be. While there are some genes that we have never been able to tag, here we include some options to try. 1) The chromatin at this locus may be very closed, and so pretreatment with something that would open the chromatin at that locus (DMSO or a specific inducer if the gene is inducible) may help. 2) Consider trying to tag the other terminus. 3) The gene may be expressed too weakly to see fluorescence or the protein may be so dispersed in the cell that the fluorescence is not visible. In this case, one option is to make a transcriptional reporter by adding a T2A sequence between the endogenous protein and the fluorescent protein and adding an NLS to the fluorescent protein to concentrate the fluorescent protein signal in the nucleus. Another option is to use a brighter fluorescent protein or multiple copies of the fluorescent protein. You can generally assess if there is correct tagging but weak fluorescence by sorting the relatively brighter cells and screening those colonies by PCR to see if the DNA sequence is or is not inserted at the locus—if the correct DNA is there but you do not see fluorescence, it is likely too dim.
Your tagged gene seems to have perturbed function	Consider tagging the other terminus, adding a longer/different linker, or adding a T2A so it becomes a transcriptional reporter. You could also investigate smaller tags.
